# Off-Label Uses of Abrocitinib: Review of Emerging Therapeutic Applications beyond Atopic Dermatitis

**DOI:** 10.3390/life14091127

**Published:** 2024-09-06

**Authors:** George G. Mitroi, George F. Mitroi, Oana Maria Ică, Florin Anghelina, Mircea Sorin Ciolofan, Mihaela Roxana Mitroi

**Affiliations:** 1 Department of Dermatology, Faculty of Medicine, University of Medicine and Pharmacy of Craiova, 200349 Craiova, Romania; 2 Department of Urology, Faculty of Medicine, University of Medicine and Pharmacy of Craiova, 200349 Craiova, Romania; 3 Department of Otorhinolaryngology, Faculty of Medicine, University of Medicine and Pharmacy of Craiova, 200349 Craiova, Romania

**Keywords:** Abrocitinib, off-label, Janus Kinase 1 (JAK1), dermatology

## Abstract

Abrocitinib, an oral small-molecule Janus Kinase 1 (JAK1) inhibitor, is primarily approved for treating moderate-to-severe atopic dermatitis (AD) in adults and adolescents aged 12 and older. This review examines the emerging off-label uses of Abrocitinib. We identified 37 papers reporting on the use of Abrocitinib in various conditions other than AD. The most commonly reported uses were for vitiligo, prurigo nodularis, and hand eczema, with 12 cases each. There were also 10 cases of lichen sclerosus and chronic pruritus of unknown origin and 5 cases each of pityriasis rubra pilaris alopecia areata. Additionally, erythematotelangiectatic rosacea and steroid-induced rosacea were reported in four cases each. Other conditions treated with Abrocitinib were noted, but these mostly had only one or two reported cases. Interestingly, out of the 103 patients reviewed, all studies reported favorable clinical outcomes and satisfactory results, with the exception of one isolated case where Abrocitinib was used to treat erythematotelangiectatic rosacea.

## 1. Introduction

**Figure 1:**
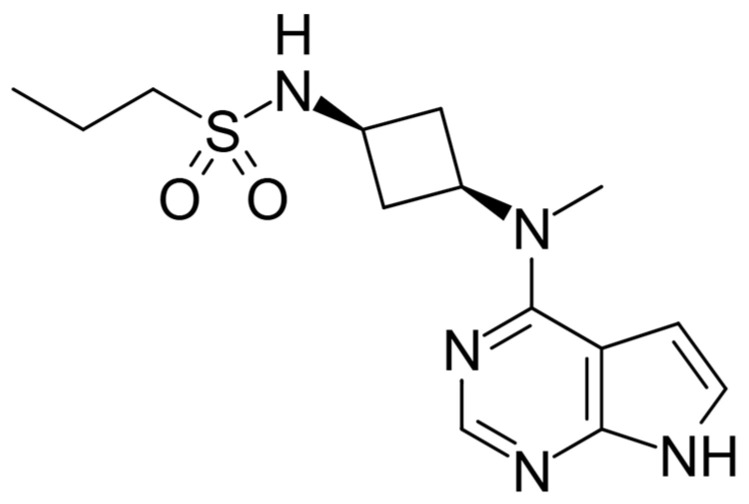
Structure of Abrocitinib drawn using ChemDraw software (version 23.1.1.3, PerkinElmer Informatics, PerkinElmer Inc., Waltham, MA, USA).

Abrocitinib is an oral small-molecule inhibitor of JAK1 used for treating moderate-to-severe AD. In 2021, it was approved in Europe for use in adults and adolescents aged 12 and older with moderate-to-severe AD who are candidates for systemic therapy [1]. It subsequently received Food and Drug Administration (FDA) approval in January 2022 [2]. Abrocitinib, having a chemical formula of C18H15FN6O2 (Figure 1), is a selective inhibitor of JAK1, demonstrating significantly greater selectivity over JAK2 (28-fold), JAK3 (>340-fold), and tyrosine kinase 2 (TYK2) (43-fold) in biochemical assays. Within cellular environments, Abrocitinib preferentially targets the cytokine-induced phosphorylation of STAT proteins mediated by JAK1-containing pairs while largely sparing JAK2/JAK2 and JAK2/TYK2 signaling.

The clinical significance of this selective inhibition remains unclear. In placebo-controlled trials lasting up to 16 weeks, Abrocitinib treatment led to dose-dependent reductions in platelet counts in patients with moderate-to-severe AD, with the lowest counts observed around week 4, followed by a trend back toward baseline levels with continued treatment [3,4].

JAK inhibitors are gaining widespread use in dermatology due to their broad potential in managing both local and systemic inflammation. Abrocitinib has recently shown promising clinical efficacy in treating various skin disorders beyond moderate-to-severe AD. In addition, the oral administration, favorable safety profile, and good tolerability of JAK inhibitors have contributed to their increasing use in treating a range of dermatologic conditions.

The JAK family consists of four types of cytoplasmic tyrosine kinases: JAK1, JAK2, JAK3, and TYK2. These kinases play a crucial role in mediating signal transduction pathways that convey signals from cell surface receptors to the nucleus. By phosphorylating tyrosine residues on specific substrates, JAKs activate downstream signaling cascades that regulate various cellular processes, including gene expression, immune response, and inflammation. Their involvement in these pathways makes them critical targets for therapeutic intervention in a range of diseases, including autoimmune disorders and cancers [5].

The JAK pathways are essential in responding to more than 50 inflammatory cytokines and play a significant role in the differentiation of naive T cells into various immune cell types, including Th1, Th2, and Th17 cells. These pathways facilitate the transmission of signals from cytokine receptors on the cell surface to the nucleus, influencing immune responses and the development of distinct T cell subsets involved in regulating inflammation and immunity [6]. These processes are closely linked to the development and progression of a broad spectrum of inflammatory and immune-mediated diseases [7].

Since gaining approval for the treatment of AD, there have been scarce reports of the off-label use of Abrocitinib for various dermatological conditions. The primary objective of this review is to identify and compile all such clinical scenarios. This paper aims to support decision making for clinicians worldwide when conventional and universally accepted treatments for a particular disease have failed, necessitating a consideration of the off-label administration of Abrocitinib.

## 2. Material and Methods

This study aims to conduct an analysis of the scientific literature regarding the off-label use of Abrocitinib in conditions beyond atopic dermatitis. We performed a review to summarize the literature on the use of Abrocitinib for treating skin disorders, excluding AD, by searching the PubMed database for studies published before 1 August 2024. The exact phrase/syntax used for the database search was “Abrocitinib”. Initially, articles were screened based on their titles, and duplicate records were removed. Any article with a title indicating the use of Abrocitinib in AD was excluded. Subsequently, papers were further screened by their abstracts, with those referring to the use of Abrocitinib in AD also being removed. Finally, full-text articles were assessed for eligibility, ensuring they focused strictly on the use of Abrocitinib for conditions other than AD. Each eligible article was then independently reviewed for data extraction.

## 3. Results and Discussion

### 3.1. Summary of Search Results

The initial search of electronic databases yielded a total of 281 articles. Following the preliminary screening, articles were excluded if their titles or abstracts indicated that Abrocitinib was used for the treatment of AD. After this filtering process, 37 articles remained and they were all included in this review. These selected studies were subsequently analyzed to assess the use of Abrocitinib in conditions other than AD, providing a comprehensive overview of its off-label applications.

Currently, Abrocitinib is approved solely for the treatment of moderate-to-severe AD. In comparison to other systemic therapies for AD, recent studies have shown that a higher proportion of patients treated with Abrocitinib achieved primary outcomes by week 4 compared to those treated with Dupilumab. Specifically, a daily dosage of 200 mg of Abrocitinib demonstrated greater efficacy than Dupilumab in adults with moderate-to-severe AD [8]. When compared to other JAK inhibitors, such as Upadacitinib and Baricitinib, no significant differences in efficacy were observed between Abrocitinib and these agents [9]. However, it is important to note that within this review, a direct comparison of Abrocitinib’s efficacy and safety to other treatments for specific diseases could not be made. Most of the studies analyzed involved an experimental off-label use of Abrocitinib, and no head-to-head trials are currently available comparing Abrocitinib with other therapies for the conditions examined in this review.

The standard dosage for Abrocitinib is either 100 mg or 200 mg once daily, though lower doses such as 10 mg or 30 mg have also been explored in clinical trials, consistently administered once per day. The drug demonstrates high absorption efficiency, with over 91% oral absorption and approximately 60% bioavailability. After oral intake, Abrocitinib reaches peak plasma levels within 1 h, and drug exposure scales linearly with doses up to 200 mg. Steady-state plasma levels are typically achieved within 48 h with daily dosing. Food intake, including high-fat meals, does not significantly alter drug exposure, so Abrocitinib can be taken with or without food. Abrocitinib and its active metabolites, M1 (3-hydroxypropyl) and M2 (2-hydroxypropyl), distribute evenly between plasma and red blood cells [10].

Among the 37 articles reviewed, 25 were single-patient case reports, 7 were case series comprising 2 to 4 patients, and 5 were brief reports involving patient cohorts ranging from 5 to 20 individuals. The findings of this review are predominantly derived from case reports and small case series, which inherently limit the generalizability of the results. Such studies lack the rigorous controls and larger sample sizes necessary to draw definitive conclusions applicable to broader populations. Consequently, while the preliminary data may be promising, it is essential to interpret these findings with caution. Further large-scale, randomized controlled trials are needed to substantiate these observations and provide more robust evidence. Until such data are available, clinicians should consider the off-label use of Abrocitinib with careful clinical judgment and awareness of its exploratory nature.

### 3.2. Abrocitinib and Vitiligo

Vitiligo is an autoimmune condition affecting the skin, in which pigment-producing melanocytes are targeted, resulting in depigmented patches that show up as white spots [11]. JAK inhibitors disrupt intracellular signaling pathways that lead to immune cell activation and the production of pro-inflammatory cytokines associated with vitiligo pathogenesis. Consequently, JAK inhibitors may help prevent additional depigmentation and promote repigmentation by facilitating melanocyte regeneration [12].

We found two papers in which Abrocitinib was used to treat vitiligo. One case report detailed a 61-year-old male with active non-segmental, acrofacial vitiligo for 2 years, who had no other significant dermatologic conditions. The patient had previously been treated with tacrolimus 0.1% ointment but was switched to Abrocitinib 100 mg daily due to inadequate results. Treatment with Abrocitinib led to significant repigmentation, with no reported side effects or recurrence of vitiligo patches after 2 months. Subsequently, the patient was transitioned back to tacrolimus 0.1% daily for maintenance, showing no relapse and continued repigmentation 4 months after stopping Abrocitinib [13]. Xu et al. reported a case series involving 11 patients with refractory progressive vitiligo, aged 26 to 59 (mean age 35.9), comprising 2 males and 9 females, with a mean disease duration of 17.7 years. All patients were treated with abrocitinib 100 mg daily for 16 weeks. Following this, 10 patients continued with abrocitinib 100 mg every other day for an additional 8 weeks, alongside narrow-band ultraviolet B therapy, resulting in favorable clinical outcomes [14]. It is noteworthy that in the case series involving 11 patients [14], the authors explicitly mention that while the improvement in vitiligo was modest, all participants reached a stable stage. In contrast, the report by Satkunanathan et al. [13] describes significant repigmentation. Among the 12 patients across both papers, 3 experienced drug-related side effects, including headache, dizziness, nausea, and gastrointestinal discomfort, but none had severe adverse effects [13,14].

### 3.3. Abrocitinib and Hand Eczema

Eczema on the hands, known as hand eczema, is a debilitating skin condition that significantly affects the quality of life and work performance of those who suffer from it. It is assumed that JAK inhibitors could be beneficial for all forms of hand eczema, although there are currently insufficient data to support this [15]. However, a study has reported favorable outcomes in patients treated with topical JAK inhibitors [16]. We identified only one case series involving 12 patients who received Abrocitinib for the treatment of hand eczema. The group consisted of seven males and five females, with a mean age of 46.3 years. All patients were treated with 100 mg of Abrocitinib daily, showing favorable clinical outcomes at the 16-week follow-up, with 90% of lesions being cured. However, the study reports that 7 out of the 12 patients experienced adverse events, which represents the highest percentage (58.3%) among all analyzed studies, regardless of the condition reported. We consider this finding to be a random occurrence, as no factors such as age, comorbidities, or the duration of treatment can be identified as contributing compared to other studies [17].

### 3.4. Abrocitinib and Prurigo Nodularis

Prurigo is a reactive skin condition marked by numerous isolated papules accompanied by intense itching [18]. Interleukin 4 (IL-4) and IL-13, which are ligands of the IL-4 receptor, are produced by T helper type 2 (Th2) cells and/or basophils. Consistently, high levels of basophils are found in the skin lesions of prurigo nodularis. Additionally, IL-31 is also produced by Th2 cells. These cytokines exert their pathophysiological effects through JAK-STAT (Janus kinase–signal transducers and activators of transcription) signaling. Thus, type 2 inflammatory cytokines derived from Th2 cells or basophils, such as IL-4, IL-13, and IL-31, may contribute to the development of prurigo nodularis via JAK-STAT signaling pathways [19]. Given these pathophysiological mechanisms, JAK inhibitors are expected to provide improvements in prurigo nodularis. We identified three studies in which Abrocitinib was administered for prurigo nodularis: two single case reports [20,21] and one case series involving 10 patients [22]. In total, 12 patients were reported, consisting of 11 females and 1 male, with a mean age of 58.8 years. Of these patients, 10 were treated with Abrocitinib 200 mg daily [22], while the remaining 2 received 100 mg daily [20,21]. Among the 12 patients, 2 achieved complete clearance of lesions within 2 months [20,21], while the remaining patients attained complete clearance within 3 months [22].

### 3.5. Abrocitinib and Chronic Pruritus of Unknown Origin

Chronic pruritus of unknown origin (CPUO) is a recently identified condition marked by persistent itching for over 6 weeks with no identifiable cause. Research suggests that both local and systemic type 2 inflammation play a role in CPUO and targeting type 2 cytokines like IL-4 and IL-13 on sensory neurons may alleviate the itch. Reports have documented the successful treatment of CPUO with Dupilumab [23,24]. However, there have been no reports of JAK inhibitors being used for CPUO since the initial study by Kwatra et al. [22]. The authors reported a case series of 10 patients, including 2 females and 8 males with a mean age of 70.7 years. All patients were treated with 200 mg of Abrocitinib daily for 12 weeks, achieving favorable clinical outcomes. However, this study also noted some of the most serious adverse effects among all analyzed studies, including two cases of scalp folliculitis, acneiform eruption, and herpes labialis. Despite these, the adverse effects are classified as mild [22].

### 3.6. Abrocitinib and Lichen Sclerosus

Lichen sclerosus is a frequently underdiagnosed inflammatory mucocutaneous condition that primarily affects the anogenital areas [25]. The condition is characterized by significant T-cell infiltration in the dermis, mainly comprising CD8+ and regulatory T-cells (Tregs), with a smaller presence of CD4+ T-cells. Although there have been reports of Baricitinib being used as a treatment for lichen sclerosus [26], the strength of recommendation and level of evidence for this treatment are still very low [25]. In this review, we identified a case series evaluating Abrocitinib for treating lichen sclerosus, involving 10 patients (7 females and 3 males) with a mean age of 35.4 years. All patients received 100 mg daily, achieving disease control after 12 weeks. Treatment was discontinued after 4 months due to favorable clinical outcomes in all cases. Only one adverse effect was reported in a single patient. Notably, this case series had one of the lowest median patient ages and the lowest frequency of adverse effects compared to the other studies reviewed [27]. Additionally, a single case report with similar results described a male patient with lichen sclerosus and plasma cell balanitis who achieved complete remission after 1 month of treatment with Abrocitinib 100 mg daily [28].

### 3.7. Abrocitinib and Pityriasis Rubra Pilaris

Pityriasis rubra pilaris (PRP) is a rare inflammatory skin disease affecting individuals of all ages, including children. PRP shares some clinical and histological features with psoriasis, for which biologics targeting tumor necrosis factor-α (TNF-α), IL-12/IL-23p40, and IL-17A have been approved. In PRP lesions, an elevated expression of pro-inflammatory cytokines (such as TNF, IL-12, and IL-23) and Th17 cytokines (particularly IL-17A and IL-22) has been observed. This supports the use of biologics targeting TNF and the IL-23-Th17 pathway for treating refractory PRP. Consequently, these biologic agents have become an increasingly preferred therapeutic option for PRP. Abrocitinib, a highly selective JAK1 inhibitor, targets multiple cytokine signaling pathways involved in the pathogenesis of pityriasis rubra pilaris PRP [29,30]. We reviewed a brief case series of five patients (two male and three female) with a mean age of 47.7 years, all treated with 100 mg of Abrocitinib daily. The treatment led to favorable clinical outcomes at the 4-week follow-up. Interestingly, one patient had previously been treated with Secukinumab and Ixekizumab, while another had tried Apremilast and Acitretin but without notable success. The positive response to Abrocitinib in these cases highlights its potential effectiveness, particularly for patients who have not responded to other therapies [31].

### 3.8. Abrocitinib and Alopecia Areata

Alopecia areata (AA) is an autoimmune disease mediated by T-cells that targets and destroys hair follicles, leading to non-scarring hair loss. This condition significantly impacts patients’ psychosomatic health and overall quality of life [32]. Preclinical studies have indicated that both type 1 and type 2 cytokines play a role in AA [33]. A recent case report showed hair regrowth after treating AA with tofacitinib, a selective inhibitor of JAK1 and JAK3 [34]. In this review, we identified three single-patient case reports and one case report involving two patients where Abrocitinib was used for AA and alopecia universalis [35,36,37,38]. Among the five patients—three females and two males—with a mean age of 26.8 years, three received 200 mg of Abrocitinib daily, while two received 100 mg daily. Notably, of all the conditions reviewed, alopecia areata had the longest median time to significant improvement or complete resolution, with a median duration of 10 months across the four papers analyzed [35,36,37,38].

### 3.9. Abrocitinib and Rosacea

Rosacea is a chronic inflammatory skin disease characterized by facial redness, papules, and flushing. The JAK-STAT signaling pathway, which regulates immune and inflammatory responses, is a promising target for treatment. JAK inhibitors, by suppressing this pathway, effectively modulate inflammatory signaling and cytokines involved in rosacea [39]. We identified three papers investigating the use of Abrocitinib for treating rosacea. These included two case series, one involving four patients with erythematotelangiectatic rosacea and the other with four patients with steroid-induced rosacea, and one single-patient case report of granulomatous rosacea. All nine patients received Abrocitinib 100 mg daily. While steroid-induced rosacea and granulomatous rosacea cases consistently reported favorable outcomes, the results for erythematotelangiectatic rosacea were inconsistent. In this group, one patient showed good clinical improvement after 4 weeks, two patients had mild improvement, and one patient showed no improvement [40,41,42]. Notably, this is the only case where Abrocitinib was ineffective across all the conditions reviewed in this paper.

### 3.10. Abrocitinib and Other Conditions

Several inflammatory and autoimmune skin conditions are being evaluated for treatment with JAK-STAT pathway inhibitors. These drugs show promise as potential alternatives to traditional immunosuppressants, such as cyclosporine, azathioprine, mycophenolate, methotrexate, and corticosteroids due to their targeted action. Unlike conventional immunosuppressants, which broadly inhibit a range of mediators, or immunobiologicals, which target specific cytokines, JAK inhibitors selectively block key cytokine signaling pathways. This targeted approach suggests that JAK inhibitors could be effective across a wide array of immune-related and inflammatory disorders [43]. In this paper, we identified several conditions treated with Abrocitinib, including pyoderma gangrenosum [44], livedoid vasculopathy [44], hidradenitis suppurativa [44], primary cutaneous lichenoid amyloidosis [45], chronic actinic dermatitis [46], bullous pemphigoid [47], tattoo granuloma with uveitis [48], granuloma annulare [49,50], psoriasis [51], post-hyaluronic-acid-filler cheek and jawline edema [52], Hailey–Hailey disease [53], Netherton syndrome [54], porokeratosis ptychotropica [55], eosinophilic pustular folliculitis [56], dissecting cellulitis of the scalp [57], mucous membrane pemphigoid [58], eruptive pruritic papular porokeratosis [59], perioral dermatitis [60], pityriasis rosea [61], necrobiosis lipoidica [62], oral lichen planus [63], and occupational airborne allergic contact dermatitis [64]. Each condition was reported in one or two cases. For more details on the reported cases, refer to Appendix A.

### 3.11. Summary of Key Findings

Abrocitinib was used to treat a diverse range of dermatological conditions. The most frequent applications were for vitiligo [13,14] and hand eczema [17], with 12 cases each. It was also utilized in 12 cases of prurigo nodularis [20,21,22] and 10 cases each of lichen sclerosus [27] and chronic pruritus of unknown origin [22]. Additional notable uses included five cases of pityriasis rubra pilaris [31] and alopecia areata [35,36,37,38] with five cases each and four cases each of erythematotelangiectatic rosacea [40] and steroid-induced rosacea [42].

The majority of studies on Abrocitinib reported using a dosage of 100 mg once daily. Exceptions to this regimen included the following:

- Granuloma annulare: one study used 150 mg daily [49], while another used 200 mg daily [50];
- Alopecia universalis: two case were treated with 200 mg daily [37,38];
- Prurigo nodularis and chronic pruritus of unknown origin: both conditions were treated with 200 mg daily in 10 cases each [22];
- Netherton syndrome: initially 200 mg daily was used for one week, then reduced to 100 mg due to adverse effects [54];
- Necrobiosis lipoidica [62] and oral lichen planus [63]: each condition was initially treated with 200 mg daily, with the lichen planus dosage reduced to 100 mg after one week.

This indicates that while 100 mg daily was the most common dosage, varying regimens were employed based on the specific condition and patient response.

Among the 103 patients reported in the analyzed studies, 19 experienced adverse effects related to Abrocitinib treatment. Most of these adverse effects were mild in nature. Commonly reported side effects included headache, dizziness, nausea, gastrointestinal discomfort, acneiform eruptions, sore throat, stomach ache, and nasal congestion [12,13,16,17,33,45]. These effects were generally transient and managed with symptomatic treatments. Less frequent adverse effects reported by individual patients included acne, blurred vision, herpes labialis, and scalp folliculitis. Each of these was observed in a single patient and typically resolved with standard care. Importantly, no cases were identified where adverse effects necessitated the discontinuation of Abrocitinib treatment. This indicates that Abrocitinib was well tolerated overall, with adverse effects being manageable and not leading to treatment cessation. The findings underscore Abrocitinib’s favorable safety profile as reported across the studies.

Overall, treatment with Abrocitinib was highly effective across various dermatological conditions, frequently resulting in the complete resolution of lesions. In most cases, therapy was discontinued upon achieving full clearance of the targeted lesions, as the primary goal of treatment was met. Notably, there was only one instance where Abrocitinib was discontinued due to its failure to produce any beneficial effects, in a patient diagnosed with erythematotelangiectatic rosacea. Detailed findings and patient outcomes are shown in Appendix A.

In all the analyzed studies, Abrocitinib was consistently administered as a once-daily dose, with patients receiving either 100 mg, 150 mg, or 200 mg per day. Across all cases, regardless of the dosage, the drug was administered solely once daily. Therefore, in the accompanying table, whenever a dosage of 100 mg, 150 mg, or 200 mg is indicated, it universally refers to a single daily dose. Of note, in all the cases analyzed, Abrocitinib was not the first-line treatment option. All patients had a significant medical history involving various prior therapies and a long-standing progression of their underlying condition. This is unsurprising, as Abrocitinib was administered off-label in every instance.

Abrocitinib has also been reported for potential use in treating SARS-CoV-2 (Severe Acute Respiratory Syndrome Coronavirus 2) due to its JAK1 inhibitory effects that may modulate immune responses. Further research is needed to confirm its effectiveness and safety for this purpose [65].

For the column titled “Concomitant Therapies with Abrocitinib”, specific concomitant therapies are explicitly listed wherever mentioned by the authors. In instances where “none” is recorded, this denotes that the authors explicitly stated the absence of concomitant therapies. Conversely, in cases where “N/A” (not applicable or not specified) is indicated, this reflects situations in which the authors did not provide information regarding concomitant therapies or where no clear statement was made concerning their presence or absence.

In the “Adverse Effects” column, the same criteria as previously described apply. The term “none” indicates that the authors explicitly reported the absence of adverse effects (AEs). In contrast, for cases where adverse effects were not mentioned, although it is reasonable to assume that any observed adverse effects would have been reported, the term “N/A” is used to denote instances where no information regarding adverse effects was provided.

The same principle applies to the “Subsequent Dose Reduction: Abrocitinib/Concomitant Therapies” column. Where dosage adjustments or treatment interruptions were reported, these changes are noted accordingly. The term “none” indicates that the authors explicitly reported no dosage modifications. In cases where no statement regarding dose reduction, interruption, or ongoing treatment was provided, the term “N/A” is used to reflect the absence of such information.

It is important to note that Abrocitinib is a relatively new medication, and as such, many of the cases documented are recent and reflect ongoing treatments. This implies that long-term efficacy, particularly in conditions other than AD, as well as the impact of dose reductions and interruptions, remain uncertain and are not yet well documented. Consequently, how patients respond to dose modifications or treatment discontinuation over extended periods is still unknown.

## 4. Conclusions

While this review suggests that Abrocitinib may have potential as a therapeutic option for various dermatological conditions beyond AD, it is important to acknowledge the limitations of the current evidence. The majority of data are derived from case reports and small case series, which restricts the ability to generalize these findings and draw definitive conclusions about its safety and efficacy. As such, these preliminary results should be interpreted with caution. To develop a more comprehensive understanding of Abrocitinib’s role in treating diverse skin disorders, future research should focus on conducting large-scale, randomized controlled trials. Key areas that require further investigation include the long-term efficacy and safety of Abrocitinib, as well as direct comparisons with existing standard treatments. Such studies will be crucial to validate these initial observations and to determine the broader applicability of Abrocitinib in dermatological practice. Until such studies are available clinicians should exercise careful clinical judgment when considering the off-label use of Abrocitinib, keeping in mind its exploratory status.

## Data Availability

Data are contained within the article.

## Abbreviations

Janus kinase	JAK
Atopic dermatitis	AD
Food and Drug Administration	FDA
Tyrosine kinase	TYK
Severe Acute Respiratory Syndrome Coronavirus-2	SARS-CoV-2
Interleukin	IL
T helper	Th
Regulatory T-cells	Tregs
Chronic pruritus of unknown origin	CPUO
Pityriasis rubra pilaris	PRP
Tumor necrosis factor-α	TNF-α
Alopecia areata	AA
Psoriasis Area and Severity Index	PASI
Body Surface Area	BSA
Dermatology Life Quality Index	DLQI
Hand Eczema Severity Index	HECSI
Adverse effect	AE
Not applicable or not specified	N/A

## Appendix A

**Table A1:** Overview of Abrocitinib as treatment for various skin disorders. N/A, not applicable or not specified; M, male; F, female; PASI, Psoriasis Area and Severity Index; BSA, Body Surface Area; DLQI, Dermatology Life Quality Index; HECSI, Hand Eczema Severity Index; AE, Adverse Effect.

Article	Age/Sex	Skin Condition	Abrocitinib Dosage	Concomitant Therapies with Abrocitinib	Clinical Outcome	Adverse Effects	Subsequent Dose Reduction: Abrocitinib/Concomitant Therapies
Satkunanathan S et al. [13]	61/M	Active non-segmental, acrofacial vitiligo	100 mg daily	N/A	Significant repigmentation, no recurrence or progression of vitiligo patches at 2 months follow-up	None	After 2 months on Abrocitinib, patient was switched back to tacrolimus 0.1% daily
Xu Z et al. [14]	11 patients (2 M/9 F) mean age 35.9	Refractory progressive vitiligo	100 mg daily; narrow-band ultraviolet B	Narrow-band ultraviolet B	Favorable clinical outcomes were noted in all patients	3/11 patients reported AEs (headache, dizzines, nausea, gastrointestinal discomfort)	Decrease in Abrocitinib after 16 weeks in 10/11 patients at 100 mg every 2 days
Li Y et al. [17]	12 patients (7 M/5 F); mean age 46.3 ± 14.1	9 patients had chronic recurrent vesicular hand eczema; 3 patients had atopic hand eczema	100 mg daily	None	All patients reached HECSI-90 at 16 weeks follow-up	7/12 patients experienced AEs, with three having multiple AEs Nausea was the most common AE (*n* = 4), followed by headache (*n* = 3). Additional AEs included acne, dizziness, and blurred vision, each reported by one patient	None
Vander Does A et al. [20]	62/F	Prurigo nodularis	100 mg daily	Ruxolitinib Triamcinolone topical for 2 months	Complete clearance after 2 months	None	None
Sun F et al. [21]	56/M	Prurigo nodularis	100 mg daily	N/A	Almost complete clearance after 2 months	N/A	None
Kwatra SG et al. [22]	10 female patients, mean age 58.6	Prurigo nodularis	200 mg daily	None	Favorable clinical outcome in all cases	4 cases of headache, nausea, acneiform eruption, sore throat, and nasal congestion	Treatment discontinued after 12 weeks in all cases according to the study design
	10 patients (2 F/8 M), mean age 70.7	Chronic pruritus of unknown origin				2 cases of scalp folliculitis, acneiform eruption, and herpes labialis	
Bao C et al. [27]	10 patients (7 F/3 M) Mean age 35.4 years	Lichen sclerosus	100 mg daily	None	All patients achieved disease control at week 12	Elevation of platelet count and total cholesterol in one patient in week 12	Treatment was discontinued after 4 months following favourable clinical outcomes in all cases
Xiong X et al. [28]	50/M	Plasma cell balanitis and lichen sclerosus	100 mg daily	0.03% tacrolimus ointment, 0.05% fluticasone propionate ointment, and 0.1% esacridine solution	Complete remission after 1 month	None	None
Li Y et al. [31]	5 patients (2 M/3 F) Mean age 47.7	Pityriasis rubra pilaris	100 mg daily	None	Baseline average PASI and BSA scores were 24.8 ± 19.61 and 39.6 ± 33.41, respectively. By week 4, these scores decreased to 4.6 ± 4.03 and 13.6 ± 11.89 The average DLQI score, initially 14.4 ± 4.63, reduced to 5.0 ± 1.9 at week 4	None	None
Bennett M et al. [35]	33/M	Alopecia areata	100 mg daily or 200 mg daily/100 mg or 200 mg daily	N/A	Complete remission in 14 weeks	N/A	N/A
	46/F	Alopecia areata AD			Complete remission after 24 weeks		
Huang J et al. [36]	11/M	Alopecia areata	100 mg daily	N/A	Significant improvement in 4 months	None	Ongoing treatment
Zhang J et al. [37]	30/F	Alopecia universalis	100 mg daily for 2 months, dose was subsequently increased to 200 mg once daily	N/A	Significant hair regrowth after 6 months	None	100 mg once daily after the 2 months in which she received 200 mg once daily
Zhao J et al. [38]	14/F	Alopecia universalis AD	200 mg daily	N/A	Complete clearance after 2 years	N/A	Ongoing treatment
Zhang T et al. [40]	37/F	Erythematotelangiectatic rosacea	100 mg daily	N/A	Significant improvement after 4 weeks	N/A	Abrocitinib reduced to 100 mg once every 4 days after 16 weeks of treatment
	41/F				Mild improvement		Medication discontinued due to hepatitis B
	38/F				Mild improvement		Patient lost to follow-up
	35/F				No improvement		Patient stopped the treatment
Ren M et al. [41]	53/F	Granulomatous rosacea	100 mg daily	N/A	Significant improvement at 20 weeks follow-up	None	Abrocitinib reduced after 20 weeks at 100 mg once weekly
Xu B et al. [42]	55/F	Steroid-induced rosacea	100 mg daily	None	Significant improvement in all cases	None	Lost to follow-up
	35/F			Topical azelaic acid gel and a skin barrier protector			Patient discontinued Abrocitinib and was maintained on topical azelaic acid gel after 6 weeks
	35/F						None
	35/F						
Chen P et al. [44]	16/M	Pyoderma gangrenosum	100 mg daily	Cyclosporine A 50 mg twice daily	Significant improvement afer 4 weeks	None	Cyclosporine A was discontinued after 4 weeks
	31/F	Livedoid vasculopathy		None	Complete remission after 6 weeks		Abrocitinib 100 mg every 2 days after 6 weeks
	17/M	Hidradenitis suppurativa		Doxycycline 100 mg twice daily	Almost complete clearance after 6 weeks	N/A	Doxycycline discontinuation after 2 weeks; Abrocitinib 100 mg once every 2 days after 6 weeks
Bai J et al. [45]	53/F	Primary cutaneous lichenoid amyloidosis	100 mg daily	N/A	Significant improvement in clinical lesions and substantial reduction in subjective symptoms at 4 months follow-up	None	Abrocitinib 100 mg every 3 days at 4 months follow-up
	59/M				Significant improvement in clinical lesions and substantial reduction in subjective symptoms at 8 weeks follow-up		Abrocitinib 100 mg every 2 days at 10 weeks follow-up
Jin X et al. [46]	70/M	Chronic actinic dermatitis	100 mg daily	N/A	Complete clearance in 6 weeks	None	Abrocitinib 100 mg twice a week after 6 weeks
Jiang W et al. [47]	52/F	Bullous pemphigoid Psoriasis	100 mg daily	60 mg methylprednisolone daily	Complete clearance	None	Reduction to 2 mg methylprednisolone daily after 1 month
	83/M	Bullous pemphigoid		4 mg methylprednisolone			None
Yang Y et al. [48]	41/M	Tattoo granuloma with uveitis	100 mg daily	Prednisone 30 mg daily with pranoprofen eye drops for uveitis	Complete clearance after 5 weeks	None	Prednisone was discontinued after 15 weeks since Abrocitinib was started
Liu W et al. [49]	29/F	Granuloma annulare	150 mg daily	N/A	Significant improvement with no new lesions at 6 weeks follow-up	None	Ongoing treatment
Michels A et al. [50]	77/F	Granuloma annulare	200 mg daily	N/A	Lesions faded within 2 weeks and disappeared within 3 months	Nausea and herpes labialis during the first week of treatment	Abrocitinib reduced to 100 mg daily after 2 weeks because of AEs; after 8 more weeks, the treatment was stopped
Mao J et al. [51]	69/F	Psoriasis	100 mg daily	None	Marked remission of skin lesions	None	Treatment was intrerrupted after 12 weeks
Lopez MHP et al. [52]	55/F	Post-zygomatic arch hyaluronic acid filler: cheek and jawline edema at 6 weeks.	100 mg daily	Fexofenadine 180 mg twice daily Fluticasone 0.0005% ointment	Complete clearance in 2 months	None	None
Li Y et al. [53]	41/F	Hailey–Hailey disease	100 mg daily	Topical zinc oxide oil	Favorable outcome at week 4 Complete clearance in 4 months	None	Ongoing treatment
Zheng CC et al. [54]	28/F	Netherton syndrome	200 mg daily	N/A	Favorable clinical outcome at 3 months follow-up	Nausea	Abrocitinb dose was decreased to 100 mg daily after 1 week of treatment due to AEs
Zhang X et al. [55]	45/M	Porokeratosis ptychotropica	100 mg daily	N/A	Improvement after 1 month Favorable outcome at 4 months follow-up	N/A	None
Cai L et al. [56]	50/M	Eosinophilic pustular folliculitis	100 mg daily	None	Improvement at 4 weeks follow-up Almost complete clearance after 6 months	None	Treatment was stopped after 7 months due to almost complete resolution of symptoms
	40/F				Improvement in lesions after 1 week of treatment, rebound after treatment stop after 8 weeks Abrocitinib was reintroduced with good results at 12 weeks follow-up		Treatment is ongoing
Jin S et al. [57]	27/M	Dissecting cellulitis of the scalp	100 mg daily	N/A	Improvement after 4 months Favorable clinical outcome at 1 year follow-up	None	Abrocitinib was reduced to 100 mg twice weekly after 1 year
Teng Y et al. [58]	62/F	Mucous membrane pemphigoid	100 mg daily	N/A	Significant improvement after 3 days Complete clearance in 4 weeks	None	Abrocitinb was reduced to 100 mg once every 2 days after 4 weeks of treatment and discontinued 2 months later
Xia J et al. [59]	75/M	Eruptive pruritic papular porokeratosis	100 mg daily	None	Favorable outcome at 1 month follow-up	None	Ongoing treatment
Teng Y et al. [60]	26/F	Perioral dermatitis	100 mg daily	None	Clinical improvement after 1 day Complete clearance after 2 weeks	None	Treatment was stopped after 12 weeks with no sign of rebound
Wu H et al. [61]	25/F	Pityriasis Rosea	100 mg daily	N/A	Significant improvement after 2 days; Complete clearance in 14 days	N/A	Treatment was stopped after 14 days
Arnet L et al. [62]	53/F	Necrobiosis lipoidica	200 mg daily	N/A	Favorable clinical outcome	Stomach ache	Abrocitinib was reduced to 100 mg daily after 12 weeks
Solimani F et al. [63]	58/M	Oral lichen planus	200 mg daily	None	Favorable clinical outcome after 12 weeks	None	Abrocitinib was reduced to 100 mg daily after 1 week
Baltazar D et al. [64]	37/M	Occupational airborne allergic contact dermatitis	100 mg daily	None	Complete clearance in 8 weeks	None	None
